# Preparation of a ε-caprolactonic diterpenoid derivate by unexpected oxidative cleavage/lactonization of 2-oxoaustroeupatol

**DOI:** 10.1007/s13659-022-00343-2

**Published:** 2022-06-01

**Authors:** Pablo A. Chacón-Morales, Juan M. Amaro-Luis, Luis Beltrán Rojas Fermín, Rémi Jacquet, Denis Deffieux, Laurent Pouységu, Stéphane Quideau

**Affiliations:** 1 Natural Products Laboratory, Department of Chemistry, Faculty of Science, University of Los Andes, Mérida, 5101 Venezuela; 2 Research Institute, Faculty of Pharmacy and Bioanalysis, University of Los Andes, Mérida, 5101 Venezuela; 3 ISM (CNRS-UMR 5255), Univ. Bordeaux, 351 cours de la Libération, 33405 Talence Cedex, France

**Keywords:** Oxidative cleavage, Austroeupatol, NaIO_4_, IBX, Lactonization, NMR

## Abstract

From aerial parts of *Austroeupatorium inulifolium* was isolated the *ent*-*nor*-furano triol labdane austroeupatol **1**. The compound **1** was treated with IBX showing an unexpected selectivity at the potentially oxidizable sites of the substrate yielding the 2-oxoaustroeupatol (**2**) and 2,19-dioxoaustroeupatol (**3**). The treatment of **2** with sodium periodate yields a heterocyclic derivative (ε-caprolactone derivate **4**) formed by oxidative cleavage and unexpected intramolecular attack of the hydroxymethylene (C-19) oxygen to the ketonic carbon (C-2). A plausible mechanistic pathway for the obtention of compound **4** is proposed.

**Graphical Abstract:**

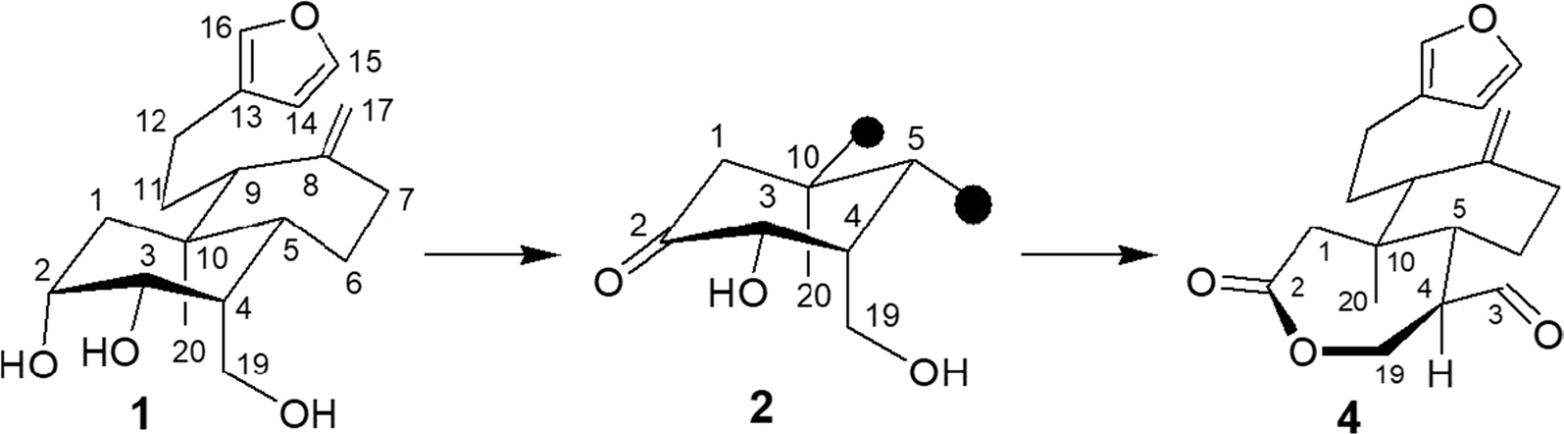

**Supplementary Information:**

The online version contains supplementary material available at 10.1007/s13659-022-00343-2.

## Introduction

Furano diterpenoids of labdane-type are representative products of *Eupatorium inulifolium* [1–3], now named *Austroeupatorium inulifolium* [4]. The substantial biological activity of these metabolites is responsible for the interest in developing methods for total synthesis of these compounds from terpenoids and chemical modifications of several available metabolites [5]. Austroeupatol (**1**) is an *ent*-*nor*-furano triol of labdane-type obtained in good yield from aerial parts of *Austroeupatorium inulifolium* [1, 2]. Due to its functionalization and relative highly abundance of compound **1** is an interesting substrate to study molecular conversions to generate novel derivatives with potential biological activity and to broaden the understanding of the structure–reactivity relationships. Here we report the generation of an unexpected ε-caprolactonic diterpenoid derivate (**4**) obtained by oxidative cleavage/lactonization of 2-oxoaustroeupatol (**2**). A plausible mechanism proposed to explain the formation of compound **4** is briefly discussed.

## Results and discussion

**Scheme 1:**
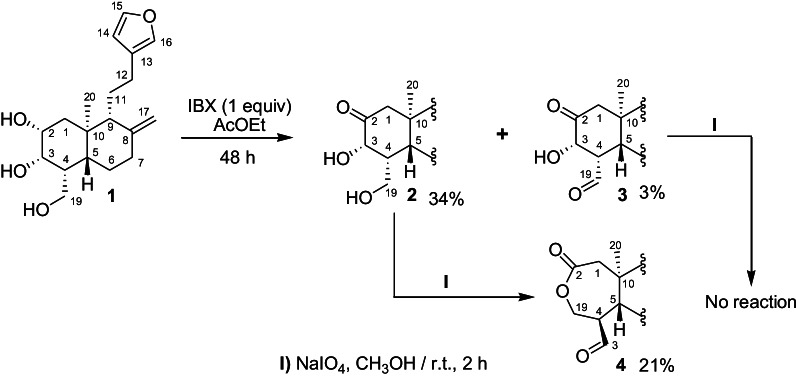
General procedure to obtain the ε-caprolactonic diterpenoid derivate **4**

The austroeupatol (**1**) was isolated as a white solid from methanol extract of the dried uncrushed leaves and stems of *A. inulifolium*. Structure **1** was established based on detailed study of the spectroscopic data (see Additional file 1). Treatment of compound **1** (300 mg) with IBX generated 2-oxoaustroeupatol **2** (34%), and 2,19-dioxoaustroeupatol **3** (3%). In previous research we have reported the isolation and description of compound **1**, and its selective oxidation with IBX [2]. The plausible explanation of IBX’s selectivity on **1**, is focused on the formation of a conformer stabilized by intramolecular hydrogen bonds [2],  which reduce the reactivity of the methylene alcohol (C-19), and methine alcohol at C-3. Upon treatment of ketone diol **2** (33 mg) with NaIO_4_, due to the absence of the glycol moiety in the substrate (**2**), the oxidative cleavage of the bond C-2/C-3 should not occur. However, the formation of a new product [**4** (21%)] was detected (Scheme 1).

**Fig. 1:**
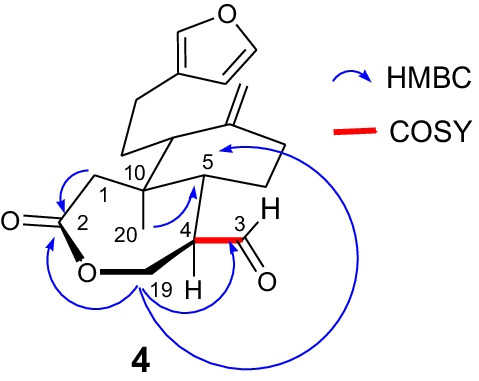
Selected key ^1^H–^1^H COSY and HMBC correlations of compound **4**

Data obtained from the analysis of ^1^H and ^13^C NMR (see Additional file 1) spectra of compound **4** allowed us to establish the molecular formula C_19_H_26_O_4_, which requires eight degrees of unsaturation (one degree of unsaturation more than the starting material **2**). This is consistent with the detection in the ^13^C NMR spectrum of two new peaks whose chemical shifts correspond to an ester [*δ*_C_: 173.3 (–O–CO–, C-2)], an aldehyde [*δ*_H_: 9.79, d, J = 2.8 Hz (–CHO, H-3); *δ*_C_: 201.6 (–CHO, C-3)], and disappearance of the ketonic peak (*δ*_C_: 210.2, C-2) of substrate **2**. The peak at δ_C_: 173.3 is attributed to oxidation of the ketonic carbon (C-2) of the starting material **2** [Δ*δ*: − 36,9 against C-2 (*δ*_C_: 210.2) of substrate **2**]. The deshielding of the oxymethylene C-19 [*δ*_H_: 4.25, d, J = 5.9 Hz (–CH_2_O–, H-19); *δ*_C_: 66.4 (–CH_2_O–, C-19); Δ*δ*: 6,2 against C-19 (*δ*_C_: 60.2) of substrate **2**] indicates that the hydroxy group of the starting material **2** was incorporated into the ester group at C-2. On the other hand, the aldehyde is generated by oxidative cleavage of the oxymethine C-3 [Δ*δ*: 123.3 against C-3 (*δ*_C_: 78.3) of substrate **2**]. The process of cleavage C-2/C-3 and cyclization C-2/-O-C-19 generated a ε-caprolactone of compound **4**. The HMBC (H-1 ↔ C-2 ↔ H-19 ↔ C-3/H-19 ↔ C-5 ↔ H-20) and COSY (H-3 ↔ H-4/H-3 ↔ H-4) correlations confirm the formation of the seven members lactone of structure **4** (Fig. 1).

**Scheme 2:**
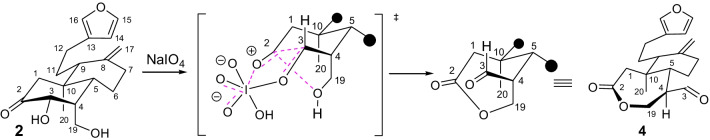
Plausible reaction mechanism proposed for the generation of ε-caprolactonic diterpenoid derivate **4**

The oxidative cleavage and subsequent formation of the heterocyclic ring can be justified by a concerted mechanism, in which periodate anion is coupled to substrate **2** through the transition state depicted in Scheme 2 and is assisted by intramolecular attack at C-2 of the C-19 hydroxy group. This hypothesis was strengthened by a pilot test in which compound **3** was used in the same reaction conditions with NaIO_4_ and no change was detected (Scheme 1); therefore, the presence of hydroxy group at C-19 is mandatory.

## Conclusion

The methanolic extract from the aerial parts of *A. inulifolium* is an important source of austroeupatol (**1**). Due to its high abundance and its high functionalization, compound **1** is an appropriate substrate to continue performing exploratory chemical reactions focused on enriching the understanding of the structure–reactivity relationships. On the other hand, we have described the procedure to obtain and elucidate a ε-caprolactonic diterpenoid derivate (**4**) from austroeupatol (**1**). Finally, a plausible mechanism to explain the formation of novel compound **4** has been proposed.

## Experimental

### General procedures

All moisture sensitive reactions were carried out in flame-dried glassware under argon atmosphere. The solvents used, tetrahydrofuran (THF) and dichloromethane (CH_2_Cl_2_), were treated immediately before use with the equipment MBRAUN Solvent Purification System. Dimethylformamide (DMF) was distilled on CaH_2_ under argon, prior to use. Evaporations were conducted under reduced pressure at temperatures less than 40 °C, unless otherwise noted. Column chromatography was carried out under positive pressure, using 40–63 µm silica gel (Merck) as adsorbent and the indicated solvents in each case. Melting points were determined with a Fisher-Johns instrument and they have not been corrected. Optical activity was measured in CHCl_3_ on 60 Hz-Steeg & Reuter G.m.b.H. polarimeter. IR spectra were recorded on a Perkin-Elmer FT-1725X spectrophotometer as film or KBr pellets. 1D and 2D NMR spectra, in the indicated solvents, were acquired with a Bruker-Avance DRX-300 instrument and calibrated using residual solvent peak as an internal standard. Mass spectra were recorded on a Hewlett-Packard Spectrometer, model 5930A (70 eV). Analytical thin layer chromatography (TLC) was developed on 0.25 mm layers of silica gel plates HF 254 (Merck) and spots were visualized by spraying with a mixture of water (235 mL), ammonium molybdate (12 g), ammonium cerium nitrate (0.5 g) and concentrated sulfuric acid (15 mL), and then heating with air flow at 100 °C for a few seconds.

### Plant material

The plant material (leaves and stems) was collected at “sector el Arenal, Municipio Libertador, Estado Mérida”. The plant was identified as *Austroeupatorium inulaefolium* by Eng. Juan Carmona Arzola, Department of Pharmacognosy and Organic Medicaments, Faculty of Pharmacy and Bioanalysis, University of Los Andes (ULA); a voucher specimen (JM Amaro-Luis & P. Chacón-Morales, No. 2333) was deposited at Natural Products Laboratory, Faculty of Science, University of Los Andes (ULA).

### Extraction and isolation procedure

The dried uncrushed leaves and stems (ca. 6 kg) was extracted with dichloromethane at room temperature for 5 min. The material was dried under hood and extracted with methanol. The solutions from both extractions were filtered and then concentrated under vacuum in a rotary evaporator at temperatures no higher than 40 °C. The extracts obtained weighed ca. 400 g (dichloromethane extract) and ca. 1616 g (methanol extract). Austroeupatol (**1**) was isolated as a white solid (9 g) in the fraction eluted with hexane–EtOAc 8:2 from methanol extract by vacuum liquid chromatography (VLC) over silica gel 60 [mp: 116–118 °C; [*α*]_D_: − 78.9 (*c *= 0.99, MeOH)]. Its IR (KBr), ν_max._ (cm^−1^): 3368 (OH); 3082 (=CH); 1642 (C=C); 1100–1250 (C–O). ^1^H NMR (300 MHz, CDCl_3_, *δ*, ppm, J/Hz): 1.2 (dd, *J* = 14.6, 3.4, H-1), 2.10 (m, H-1ʹ), 4.12 (d, *J* = 3.0, H-2), 3.77 (dd, *J* = 5.3, 3.3, H-3), 2.10 (m, H-4), 1.51 (m, H-5), 1.61 (m, H-6), 2.40 (m, H-7), 1.99 (m, H-7ʹ), 1.54 (m, H-9), 1.61–1.41 (m, H-11), 2.54 (m, H-12), 2.19 (m, H-12ʹ), 6.23 (d, *J* = 0.7, H-14), 7.32 (t, *J* = 1.5, H-15), 7.17 (s, H-16), 4.91 (s, H-17), 4.59 (s, H-17ʹ), 4.46 (t, *J* = 10.2, H-19), 3.60 (d, *J* = 8.8, H-19ʹ), 0.76 (s, H-20). ^13^C NMR (75 MHz, CDCl_3_, *δ*, ppm): 42.4 (C-1), 71.0 (C-2), 74.8 (C-3), 46.6 (C-4), 47.4 (C-5), 29.1 (C-6), 38.0 (C-7), 146.8 (C-8), 55.6 (C-9), 37.5 (C-10), 24.5 (C-11), 23.3 (C-12), 125.2 (C-13), 110.9 (C-14), 142.6 (C-15), 138.6 (C-16), 108.1 (C-17), 61.7 (C-19), 15.7 (C-20).

### Compound 2

To a stirred solution of austroeupatol **1** (300 mg, 9.36 × 10^–1^ mmol) in EtOAc (15 mL) was added IBX (315 mg, 1.1 mmol) for 48 h. The crude was purified by column chromatography, eluting with CH_2_Cl_2_/EtOAc (9:1). Yellow resin, yield: 34%. ^1^H NMR (300 MHz, CDCl_3_, δ, ppm, J/Hz): 2.55 (m, H-1), 4.46 (d, J = 7.82, H-3), 2.11 (m, H-4), 2.64 (m, H-5), 1.67 (m, H-6), 2.46 (m, H-7), 2.09 (m, H-7ʹ), 1.88 (d, J = 10.80, H-9), 1.66–1.47 (m, H-11), 2.57 (m, H-12), 2.25 (m, H-12ʹ), 6.20 (m, H-14), 7.32 (t, J = 1.5, H-15), 7.16 (s broad, H-16), 4.96 (d, J = 1.11, H-17), 4.64 (s broad, H-17ʹ), 3.44 (m, H-19), 0.76 (s, H-20). ^13^C NMR (75 MHz, CDCl_3_, δ, ppm): 50.8 (C-1), 210.2 (C-2), 78.3 (C-3) 46.4 (C-4), 51.9 (C-5), 28.3 (C-6), 37.5 (C-7), 145.4 (C-8), 54.1 (C-9), 44.6 (C-10), 24.6 (C-11), 23.1 (C-12), 124.8 (C-13), 110.7 (C-14), 143.0 (C-15), 138.9 (C-16), 108.9 (C-17), 60.2 (C-19), 15.0 (C-20).

### Compound 3

To a stirred solution of austroeupatol **1** (300 mg, 9.36 × 10^–1^ mmol) in EtOAc (15 mL) was added IBX (315 mg, 1.1 mmol) for 48 h. The crude was purified by column chromatography, eluting with CH_2_Cl_2_/EtOAc (9:1). Yellow resin, yield: 3%. ^1^H NMR (300 MHz, CDCl_3_, *δ*, ppm, J/Hz): 2.21 (d, *J* = 13.2, H-1), 2.68 (d, *J* = 13.2, H-1ʹ), 4.40 (d, *J* = 7.8, H-3), 3.13 (m, H-4), 2.29 (m, H-5), 1.72 (m, H-6), 2.48 (m, H-7), 2.04 (m, (H-7ʹ), 1.95 (d, *J* = 10.9 H-9), 1.69–1.50 (m, H-11), 2.22 (m, H-12), 2.59 (m, H-12ʹ), 6.23 (s broad, H-14), 7.35 (t, *J* = 1.5, H-15), 7.19 (s, H-16), 4.68 (s broad, H-17), 5.00 (d, *J* = 1.1, H-17ʹ), 9.63 (d, *J* = 3.06, H-19), 0.59 (s, H-20). ^13^C NMR (75 MHz, CDCl_3_, *δ*, ppm): 51.0 (C-1), 208.9 (C-2), 75.4 (C-3), 58.8 (C-4), 48.3 (C-5), 28.3 (C-6), 37.5 (C-7), 144.9 (C-8), 53.5 (C-9), 44.9 (C-10), 24.8 (C-11), 23.1 (C-12), 124.8 (C-13), 110.8 (C-14), 143.2 (C-15), 139.1 (C-16), 109.4 (C-17), 201.6 (C-19), 15.9 (C-20).

### Compound 4

To a stirred solution of compound **2** (33 mg, 1.06 × 10^–1^ mmol) in CH_3_OH (10 mL) was added sodium periodate (NaIO_4_) (23 mg, 1.08 × 10^–1^ mmol). After 2 h at room temperature, the CH_3_OH was removed in vacuo. Over the resin product was added H_2_O (5 mL) and washed with EtOAc (4 × 5 mL). The organic phase was washed with brine (5 mL) and dried over MgSO_4_. The crude was purified by column chromatography, eluting with CH_2_Cl_2_/CH_3_OH (99:1) White resin. Yield 21%. MF: C_19_H_26_O_4_. ^1^H NMR (300 MHz, CDCl_3_, *δ*, ppm, J/Hz): 2.61 (d, *J* = 13.8, 3.4, H-1), 2.85 (d, *J* = 13.8, H-1ʹ), 9.79 (d, *J* = 2.8, H-3), 2.81 (m, H-4), 1.84 (m, H-5), 1.78 (m, H-6), 2.03 (m, H-7), 2.40 (m, H-7ʹ), 2.04 (m, H-9), 1.41 (m, H-11), 1.68 (m, H-11ʹ), 2.27 (m, H-12), 2.60 (m, (H-12ʹ), 6.27 (s broad, H-14), 7.36 (s broad, H-15), 7.21 (s, H-16), 4.78 (s, H-17), 5.03 (d, *J* = 0.90, H-17′ 4.25, (d, *J* = 5.9, H-19), 0.81 (s, H-20). ^13^C NMR (75 MHz, CDCl_3_, δ, ppm): 43.6 (C-1), 173.3 (C-2), 201.6 (C-3), 53.4 (C-4), 51.3 (C-5), 25.7 (C-6), 37.1 (C-7), 145.4 (C-8), 51.1 (C-9), 39.8 (C-10), 28.4 (C-11), 23.7 (C-12), 124.6 (C-13), 110.9 (C-14), 143.2 (C-15), 139.1 (C-16), 110.2 (C-17), 66.4 (C-19), 13.3 (C-20).

### Exploratory reaction: 2,19-dioxoaustroeupatol (3) and sodium periodate

To a stirred solution of compound **3** (5 mg, 1.6 × 10^–2^ mmol) in CH_3_OH (10 mL) was added Sodium Periodate (NaIO_4_) (4 mg, 1.87 × 10^–2^ mmol). After 4 h of reaction no change was detected.

## Supplementary Information

**Additional file 1.** Reactions schemes. Additional figures S1–S21.
