# Supplementary material for: Preparation of a ε-caprolactonic diterpenoid derivate by unexpected oxidative cleavage/lactonization of 2-oxoaustroeupatol
Source: Nat Prod Bioprospect. 2022 Jun 1;12(1):20. doi: 10.1007/s13659-022-00343-2 (PMC9156645; doi:10.1007/s13659-022-00343-2)

SUPPLEMENTARY MATERIAL

Preparation of a ε-caprolactonic diterpenoid derivate by unexpected oxidative cleavage / lactonization of 2-oxoaustroeupatol

Pablo A. Chacón-Morales[a]*, Juan M. Amaro-Luis[a], Luis Beltrán Rojas Fermín[b], Rémi Jacquet[c], Denis Deffieux[c], Laurent Pouységu[c] & Stéphane Quideau[c]

[a]*Natural Products Laboratory, Department of Chemistry, Faculty of Science, University of Los Andes, Mérida, Venezuela 5101*

[b] *Research Institute, Faculty of Pharmacy and Bioanalysis, University of Los Andes, Mérida, 5101, Venezuela*
[c] Univ. Bordeaux, ISM (CNRS-UMR 5255) 351 cours de la Libération, 33405 Talence Cedex, France

Contents of Supplementary material

N°	Contents	Page
4	**Reactions schemes** …………………………………………………………….……………...	…3
5	**Figure S1.** IR spectrum (KBr) of austroeupatol **1**..	…4
6	**Figure S2.** ^{1}H NMR spectrum (300 MHz, $CDCl_3$) of austroeupatol **1**…………………………………	5
7	**Figure S3.** ^{13}C NMR spectrum (75 MHz, $CDCl_3$) of austroeupatol **1**…………………………..……	6
8	**Figure S4.** ^{1}H-^{1}H COSY spectrum ($CDCl_3$) of austroeupatol **1**……………………………………...	…7
9	**Figure S5**. HMQC spectrum ($CDCl_3$) of austroeupatol **1**………………………………………………	…8
10	**Figure S6.** HMBC spectrum ($CDCl_3$) of austroeupatol **1**……………………………………………….	…9

Figure S7. ^{1}H NMR spectrum (300 MHz, $CDCl_3$) of compound **2**……………………………………. ..10
Figure S8. ^{13}C NMR spectrum (75 MHz, $CDCl_3$) of compound **2**…………………………………….. ..11
Figure S9. ^{1}H-^{1}H COSY ($CDCl_3$) spectrum of compound **2**.. ..12
Figure S10 HMQC ($CDCl_3$) spectrum of compound **2**……………………………………………….. ..13
Figure S11 HMBC ($CDCl_3$) spectrum of compound **2**………………………………………………… ..14
Figure S12 ^{1}H NMR spectrum (300 MHz, $CDCl_3$) of compound **3**…………………………………… ..15
Figure S13. ^{13}C NMR spectrum (75 MHz, $CDCl_3$,) of compound **3**………………….……………….. ..16
Figure S14. ^{1}H-^{1}H COSY spectrum of ($CDCl_3$) of compound **3**………………………………………. ..17
Figure S15. HMQC spectrum of ($CDCl_3$) of compound **3**……………………………..……………… ..18
Figure S16 HMBC spectrum of ($CDCl_3$) of compound **3**.. ..19
Figure S17. ^{1}H NMR spectrum (300 MHz, $CDCl_3$) of compound **4**………………………..……… ..20
Figure S18. ^{13}C NMR spectrum (75 MHz, $CDCl_3$) of compound **4**…………………………………… ..21
Figure S19. ^{1}H-^{1}H COSY spectrum of ($CDCl_3$) of compound **4**………………………………………. ..22
Figure S20. HMQC spectrum of ($CDCl_3$) of compound **4**... ..23
Figure S21. HMBC spectrum of ($CDCl_3$) of compound **4**... ..24

Reaction schemes

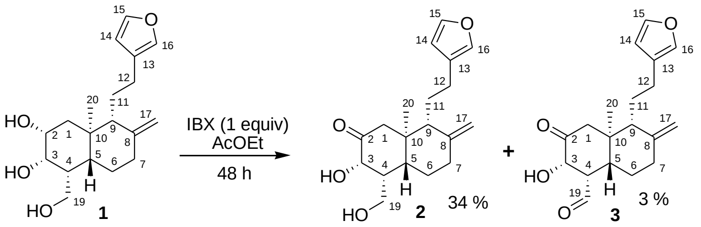

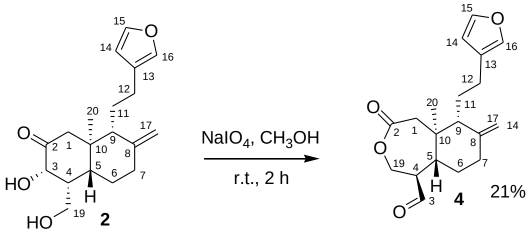

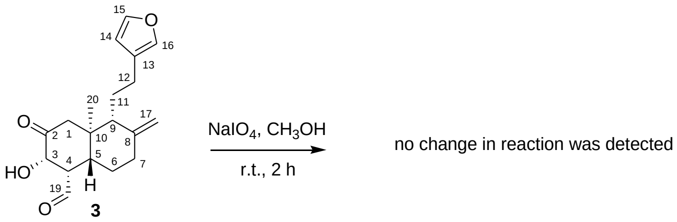

Figure S1. IR spectrum (KBr) of austroeupatol **1**

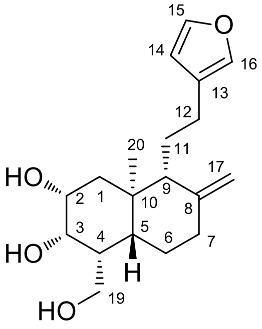

1

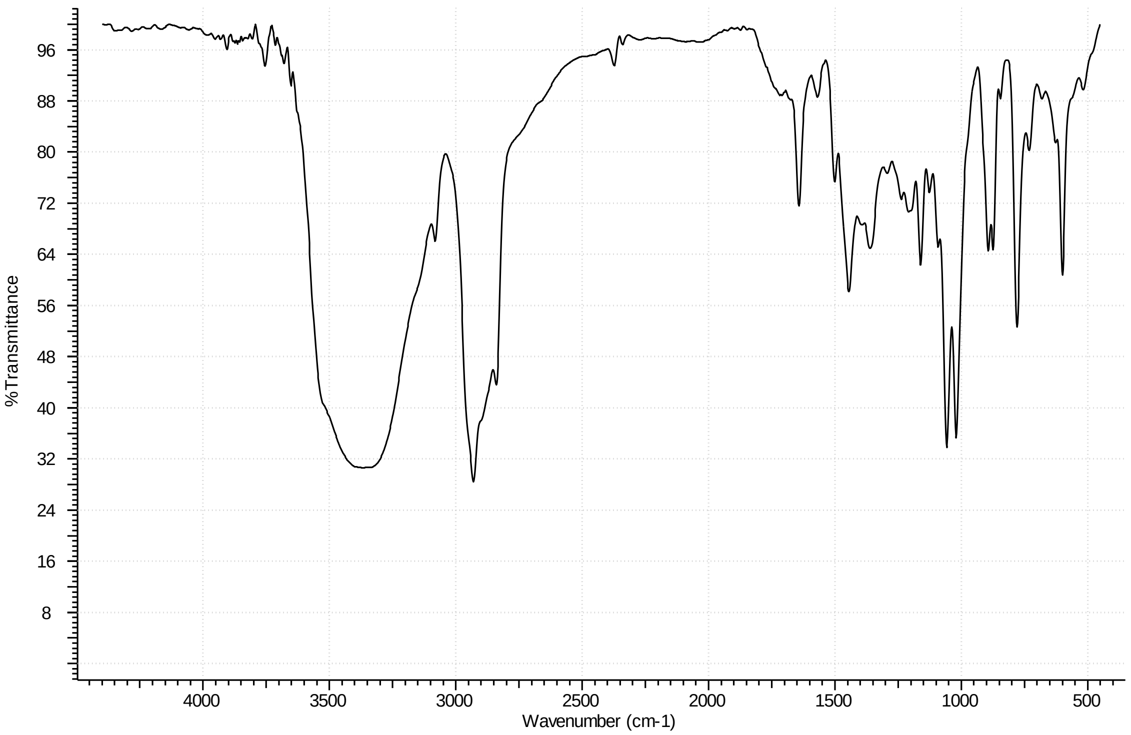

Figure S2. ^{1}H NMR spectrum (300 MHz, $CDCl_3$) of austroeupatol **1**

^{1}H NMR (300 MHz, $CDCl_3$, δ, ppm, J/Hz): 1.2 (dd, J = 14.6, 3.4, H-1), 2.10 (m, H-1'), 4.12 (d, J = 3.0, H-2), 3.77 (dd, J = 5.3, 3.3, H-3), 2.10 (m, H-4), 1.51 (m, H-5), 1.61 (m, H-6), 2.40 (m, H-7), 1.99 (m, H-7'), 1.54 (m, H-9), 1.61-1.41 (m, H-11), 2.54 (m, H-12), 2.19 (m, H-12'), 6.23 (d, J = 0.7, H-14), 7.32 (t, J = 1.5, H-15), 7.17 (s, H-16), 4.91 (s, H-17), 4.59 (s, H-17'), 4.46 (t, J = 10.2, H-19), 3.60 (d, J = 8.8, H-19'), 0.76 (s, H-20).

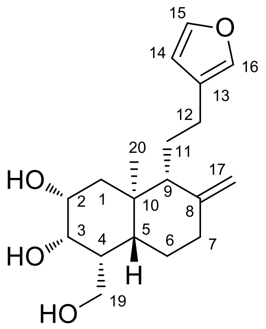

1

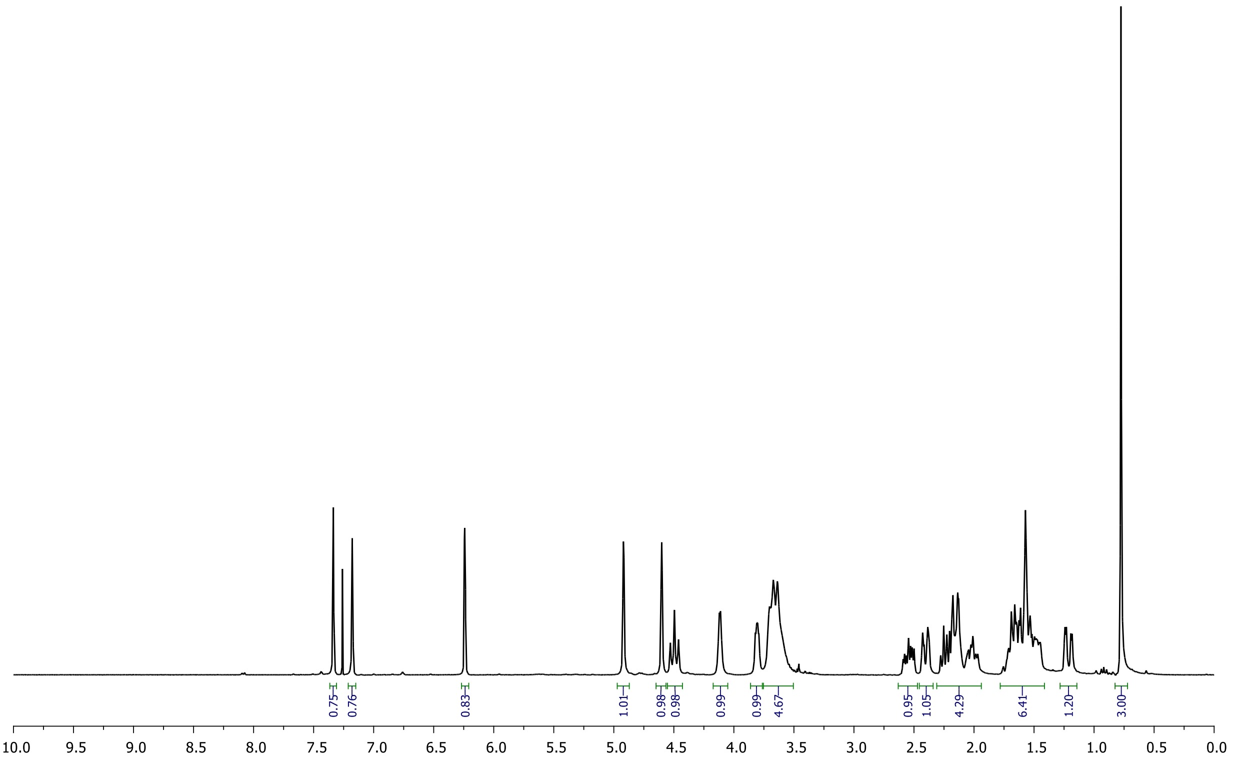

Figure S3. ^{13}C NMR spectrum (75 MHz, $CDCl_3$) of of austroeupatol **1**

^{13}C NMR (75 MHz, $CDCl_3$, δ, ppm): 42.4 (C-1), 71.0 (C-2), 74.8 (C-3), 46.6 (C-4), 47.4 (C-5), 29.1 (C-6), 38.0 (C-7), 146.8 (C-8), 55.6 (C-9), 37.5 (C-10), 24.5 (C-11), 23.3 (C-12), 125.2 (C-13), 110.9 (C-14), 142.6 (C-15), 138.6 (C-16), 108.1 (C-17), 61.7 (C-19), 15.7 (C-20).

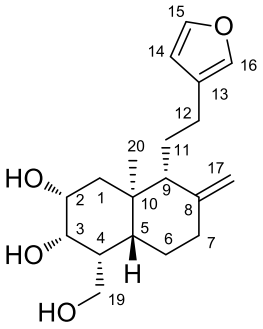

1

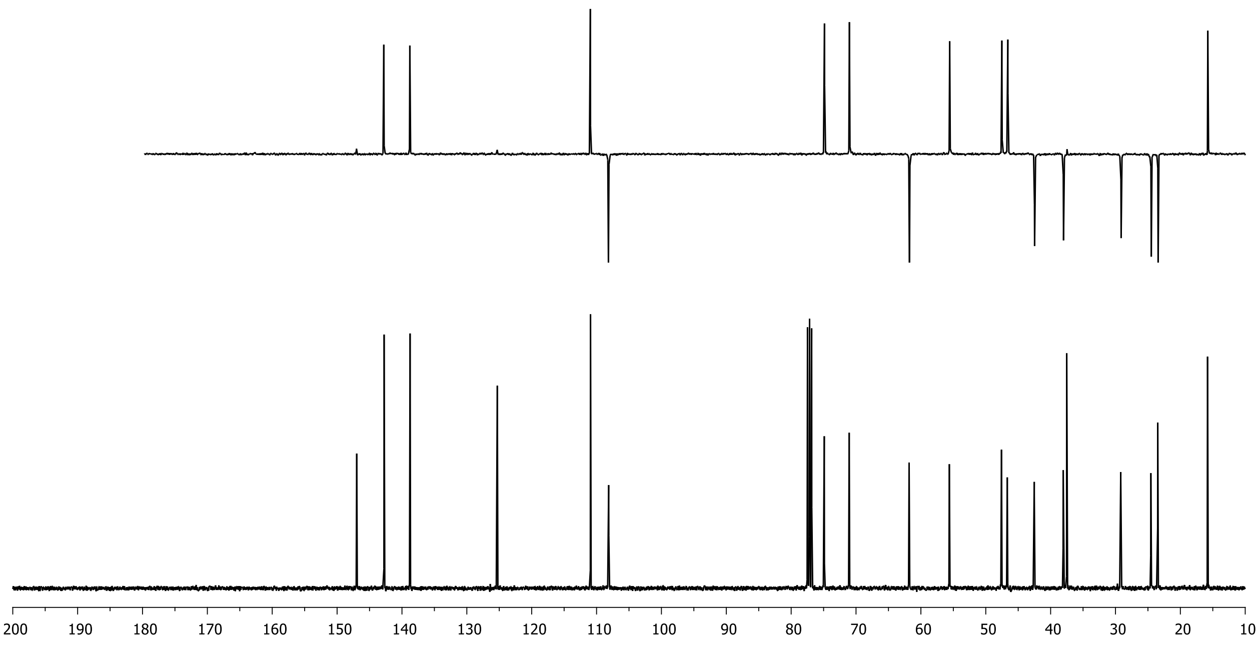

Figure S4. ^{1}H-^{1}H COSY spectrum (CDCl$_3$) of austroeupatol **1**

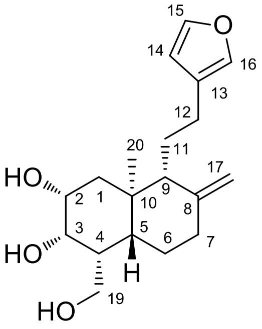

1

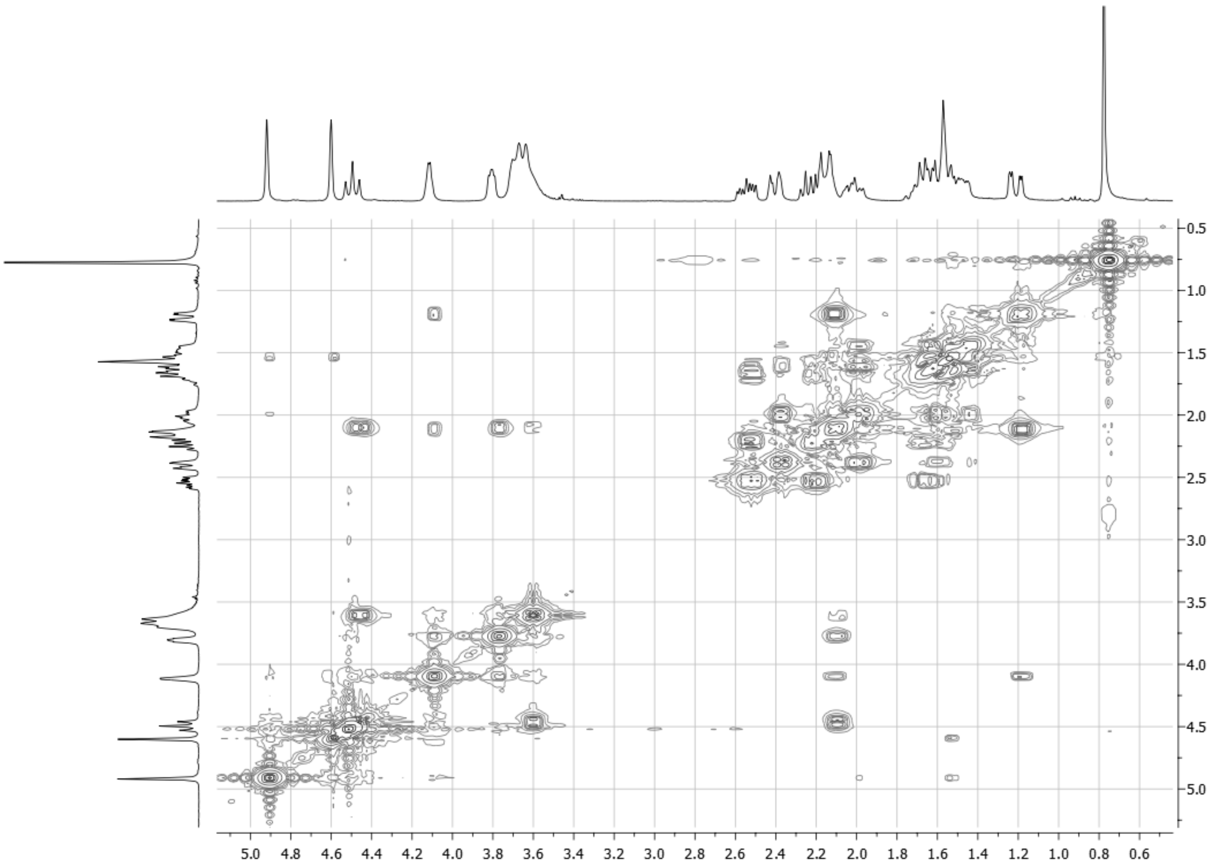

Figure S5. HMQC spectrum ($CDCl_3$) of austroeupatol **1**

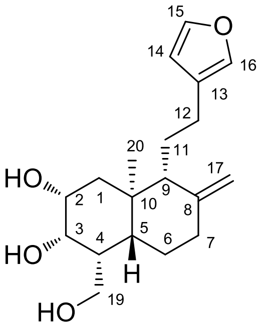

1

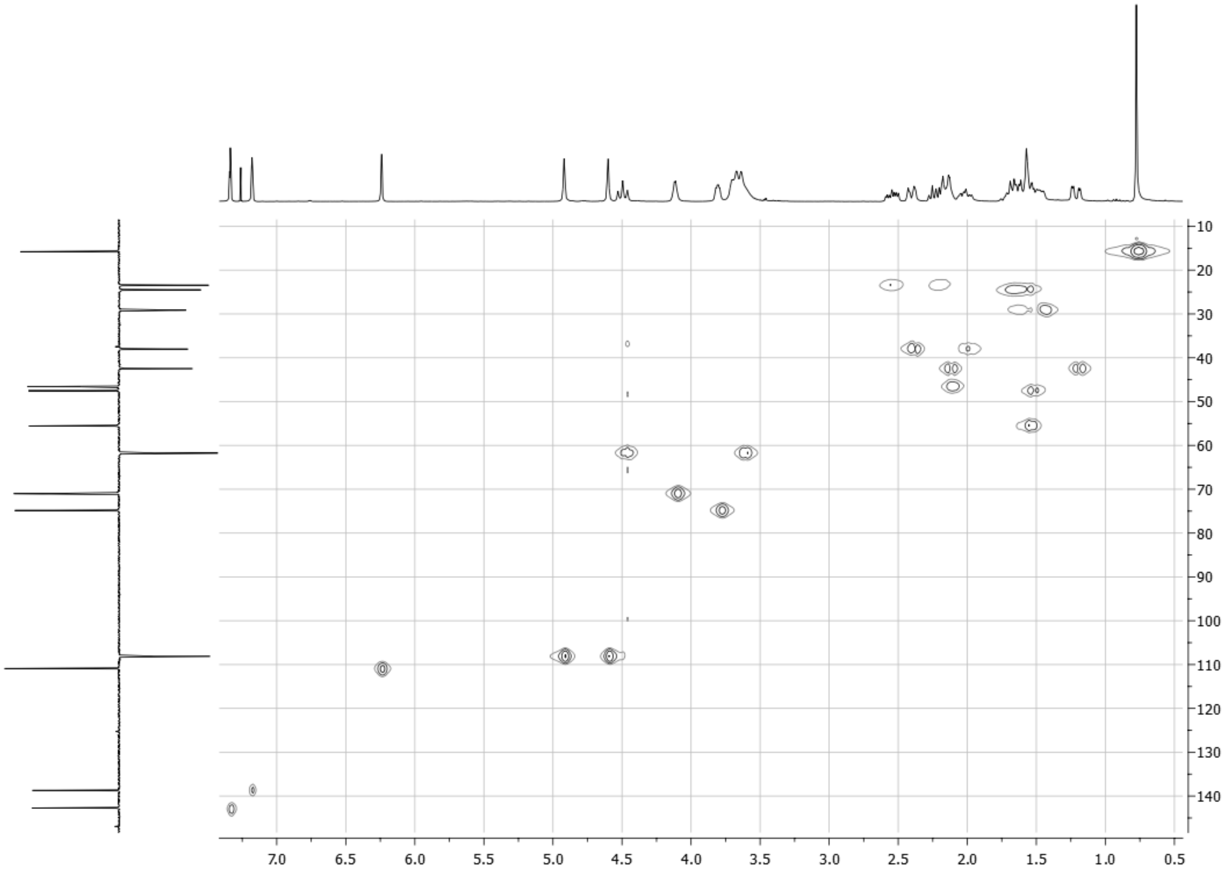

Figure S6. HMBC spectrum ($CDCl_3$) of austroeupatol **1**

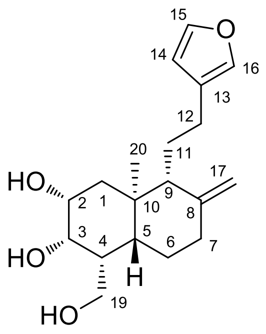

1

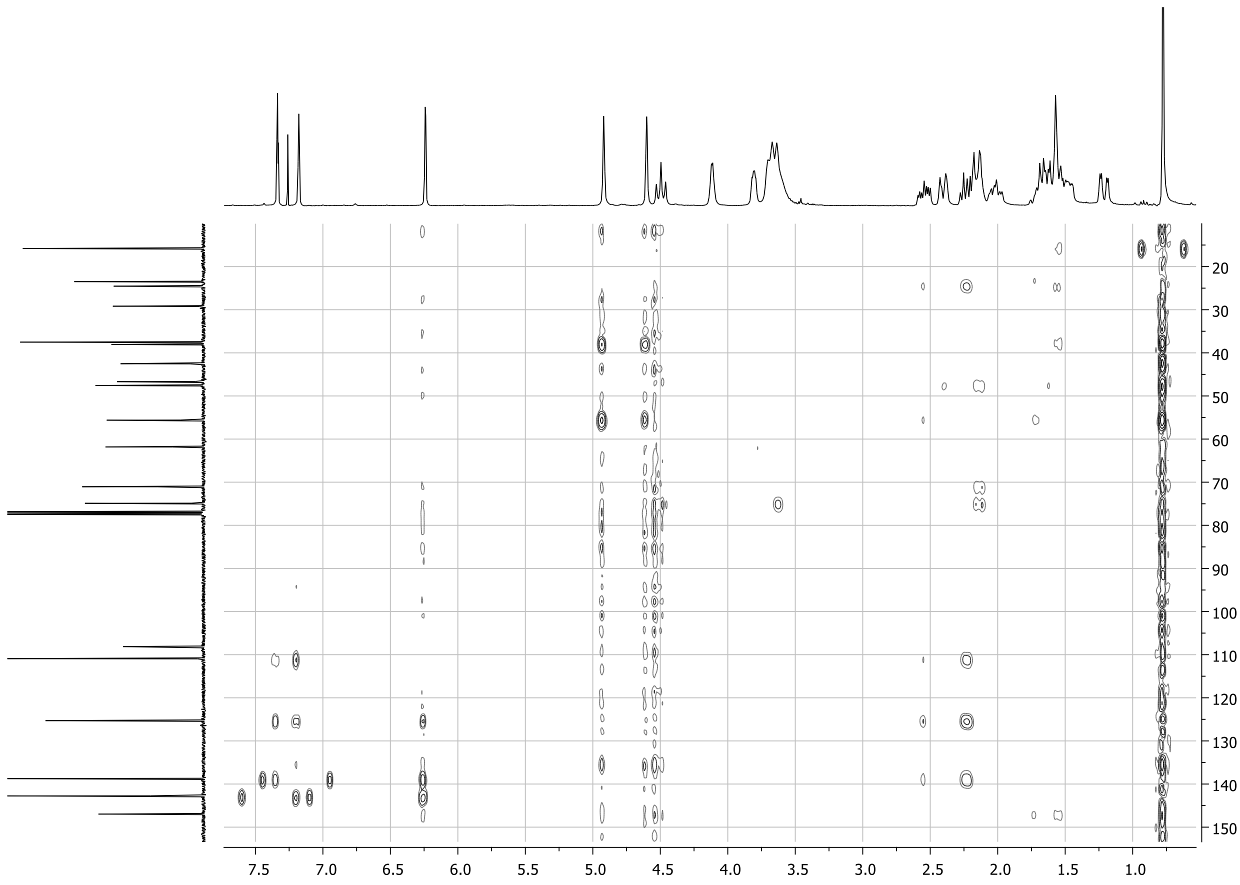

Figure S7. ^{1}H NMR spectrum (300 MHz, $CDCl_3$) of compound **2**

^{1}H NMR (300 MHz, $CDCl_3$, δ, ppm, J/Hz): 2.55 (m, H-1), 4.46 (d, J = 7.82, H-3), 2.11 (m, H-4), 2.64 (m, H-5), 1.67 (m, H-6), 2.46 (m, H-7), 2.09 (m, H-7’), 1.88 (d, J = 10.80, H-9), 1.66-1.47 (m, H-11), 2.57 (m, H-12), 2.25 (m, H-12’), 6.20 (m, H-14), 7.32 (t, J = 1.5, H-15), 7.16 (s broad, H-16), 4.96 (d, J = 1.11, H-17), 4.64 (s broad, H-17’), 3.44 (m, H-19), 0.76 (s, H-20).

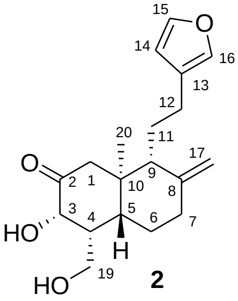

2

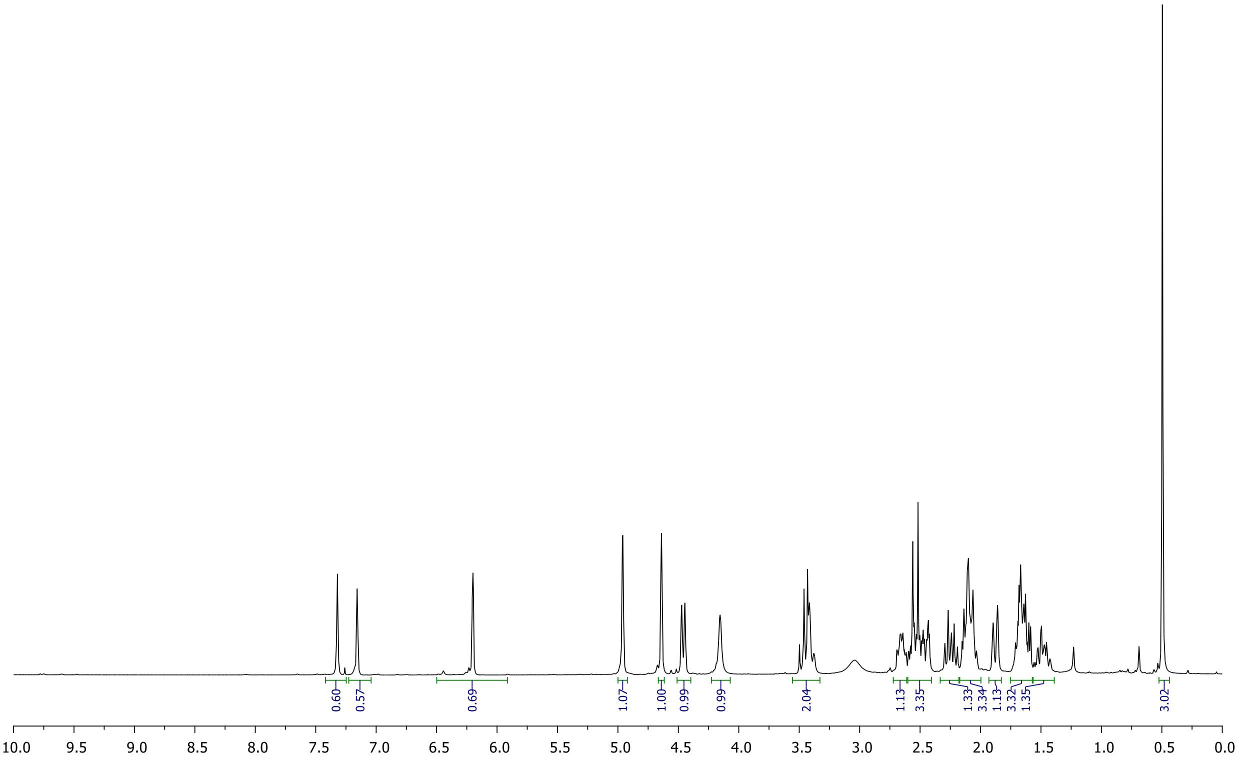

Figure S8. ^{13}C NMR spectrum (75 MHz, $CDCl_3$) of compound **2**

^{13}C NMR (75 MHz, $CDCl_3$, δ, ppm): 50.8 (C-1), 210.2 (C-2), 78.3 (C-3) 46.4 (C-4), 51.9 (C-5), 28.3 (C-6), 37.5 (C-7), 145.4 (C-8), 54.1 (C-9), 44.6 (C-10), 24.6 (C-11), 23.1 (C-12), 124.8 (C-13), 110.7 (C-14), 143.0 (C-15), 138.9 (C-16), 108.9 (C-17), 60.2 (C-19), 15.0 (C-20).

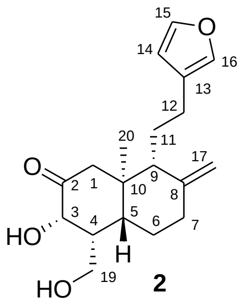

2

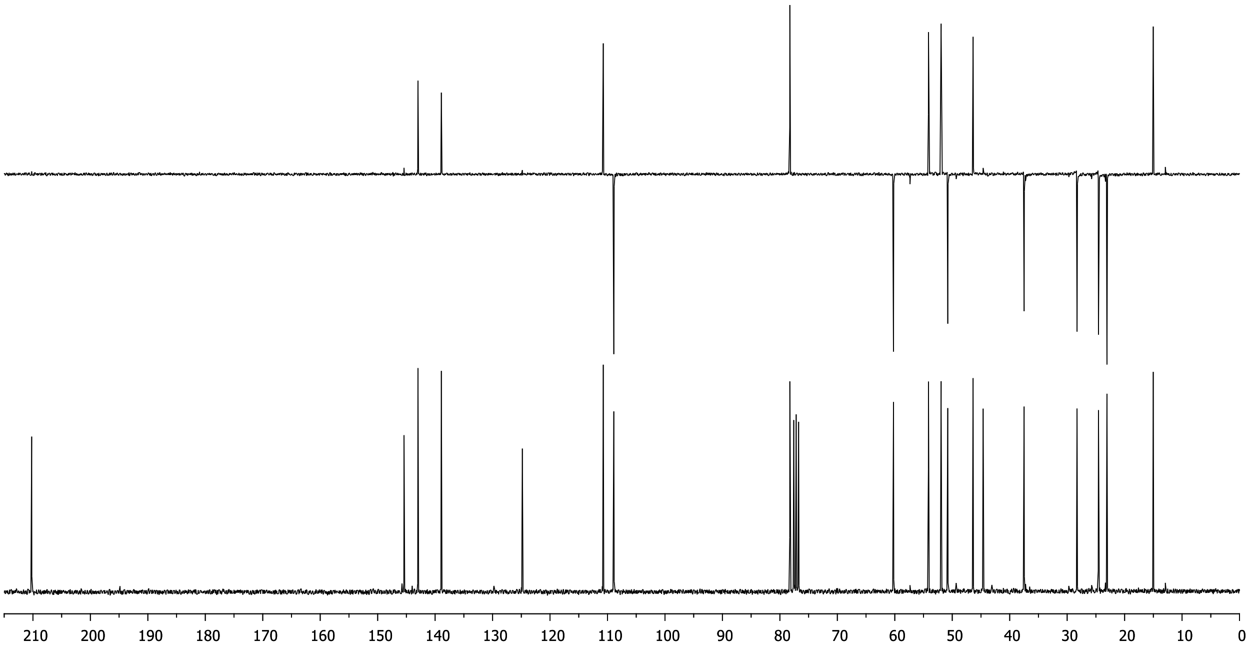

Figure S9. ^{1}H-^{1}H COSY ($CDCl_3$) spectrum of compound **2**

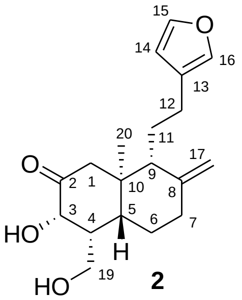

2

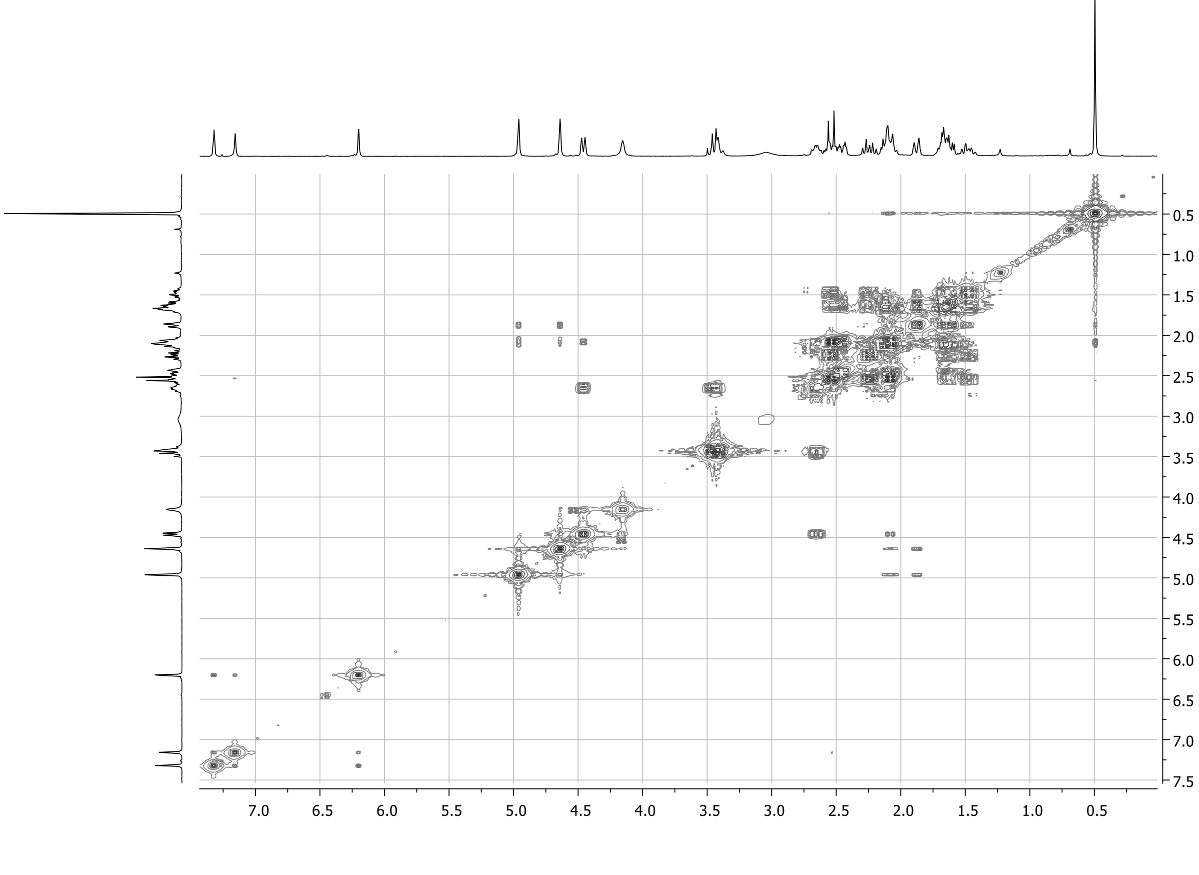

Figure S10. HMQC ($CDCl_3$) spectrum of compound **2**

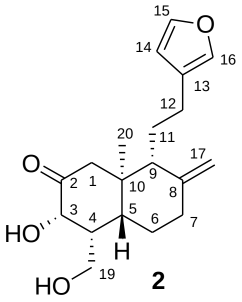

2

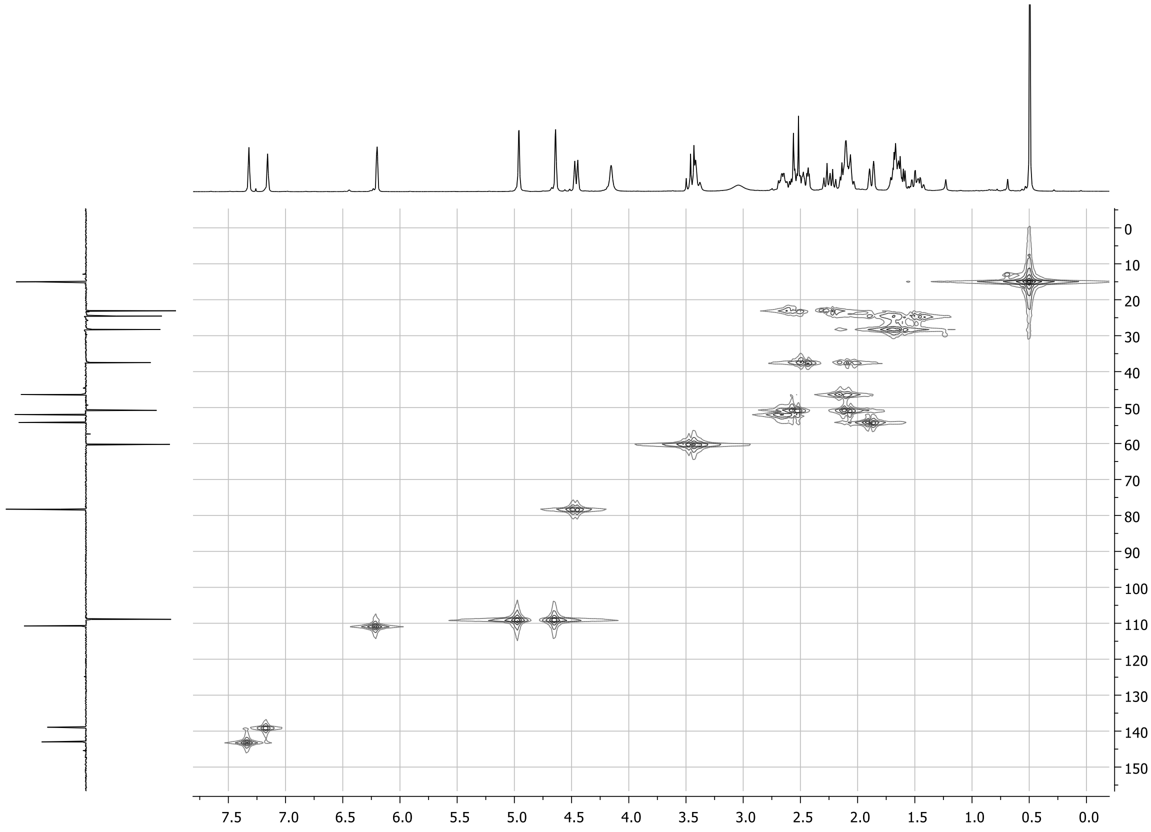

Figure S11. HMBC ($CDCl_3$) spectrum of compound **2**

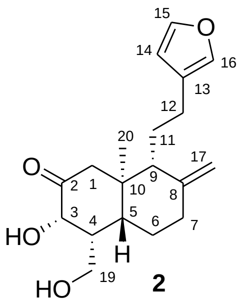

2

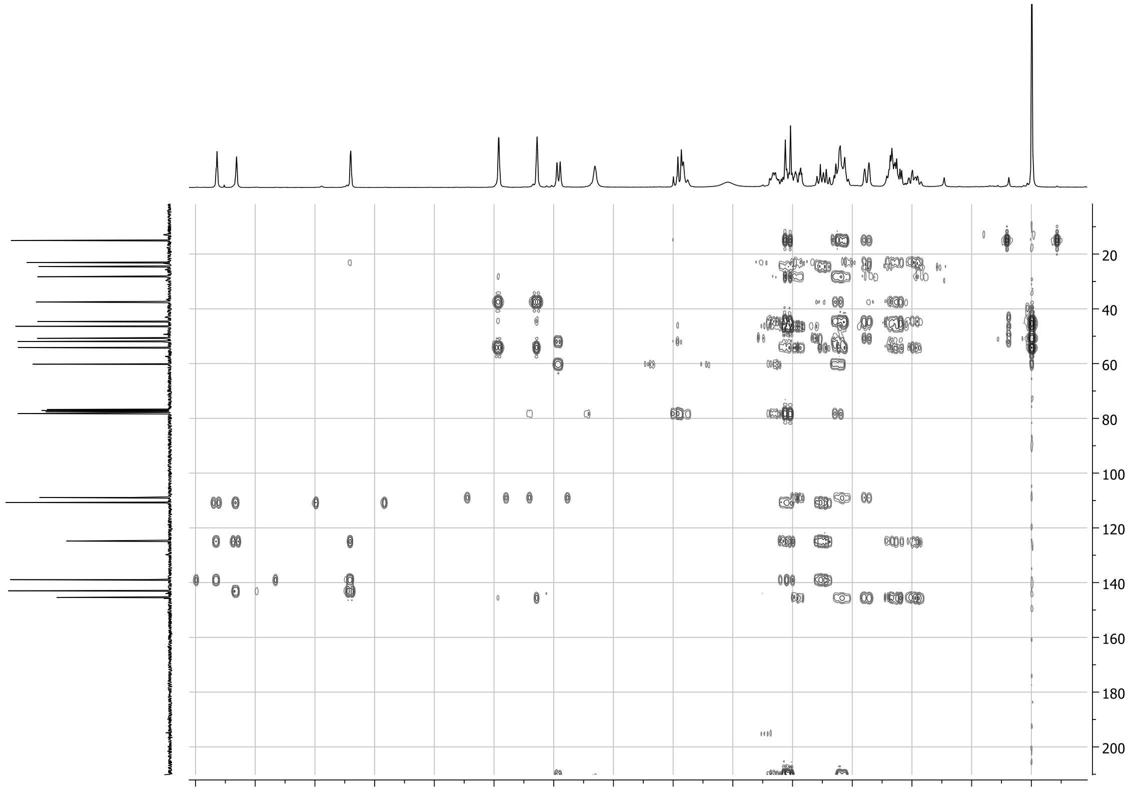

Figure S12. ^{1}H NMR spectrum (300 MHz, $CDCl_3$) of compound **3**

^{1}H NMR (300 MHz, $CDCl_3$, δ, ppm, J/Hz): 2.21 (d, J = 13.2, H-1), 2.68 (d, J = 13.2, H-1’), 4.40 (d, J = 7.8, H-3), 3.13 (m, H-4), 2.29 (m, H-5), 1.72 (m, H-6), 2.48 (m, H-7), 2.04 (m, (H-7’), 1.95 (d, J = 10.9 H-9), 1.69-1.50 (m, H-11), 2.22 (m, H-12), 2.59 (m, H-12’), 6.23 (s broad, H-14), 7.35 (t, J = 1.5, H-15), 7.19 (s, H-16), 4.68 (s broad, H-17), 5.00 (d, J = 1.1, H-17’), 9.63 (d, J = 3.06, H-19), 0.59 (s, H-20).

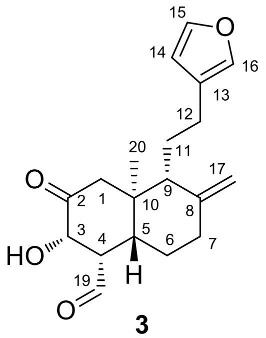

3

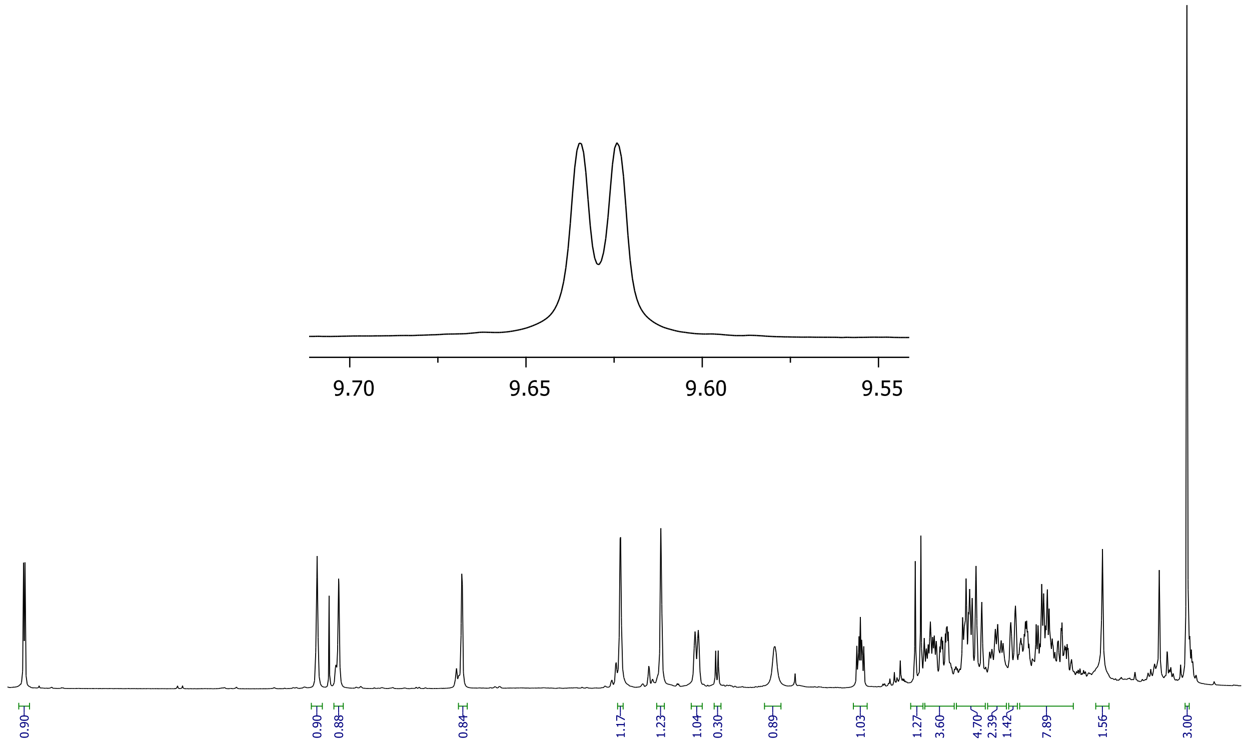

Figure S13. ^{13}C NMR spectrum (75 MHz, $CDCl_3$) of compound **3**

^{13}C NMR (75 MHz, $CDCl_3$, δ, ppm): 51.0 (C-1), 208.9 (C-2), 75.4 (C-3), 58.8 (C-4), 48.3 (C-5), 28.3 (C-6), 37.5 (C-7), 144.9 (C-8), 53.5 (C-9), 44.9 (C-10), 24.8 (C-11), 23.1 (C-12), 124.8 (C-13), 110.8 (C-14), 143.2 (C-15), 139.1 (C-16), 109.4 (C-17), 201.6 (C-19), 15.9 (C-20).

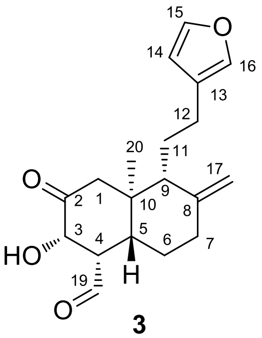

3

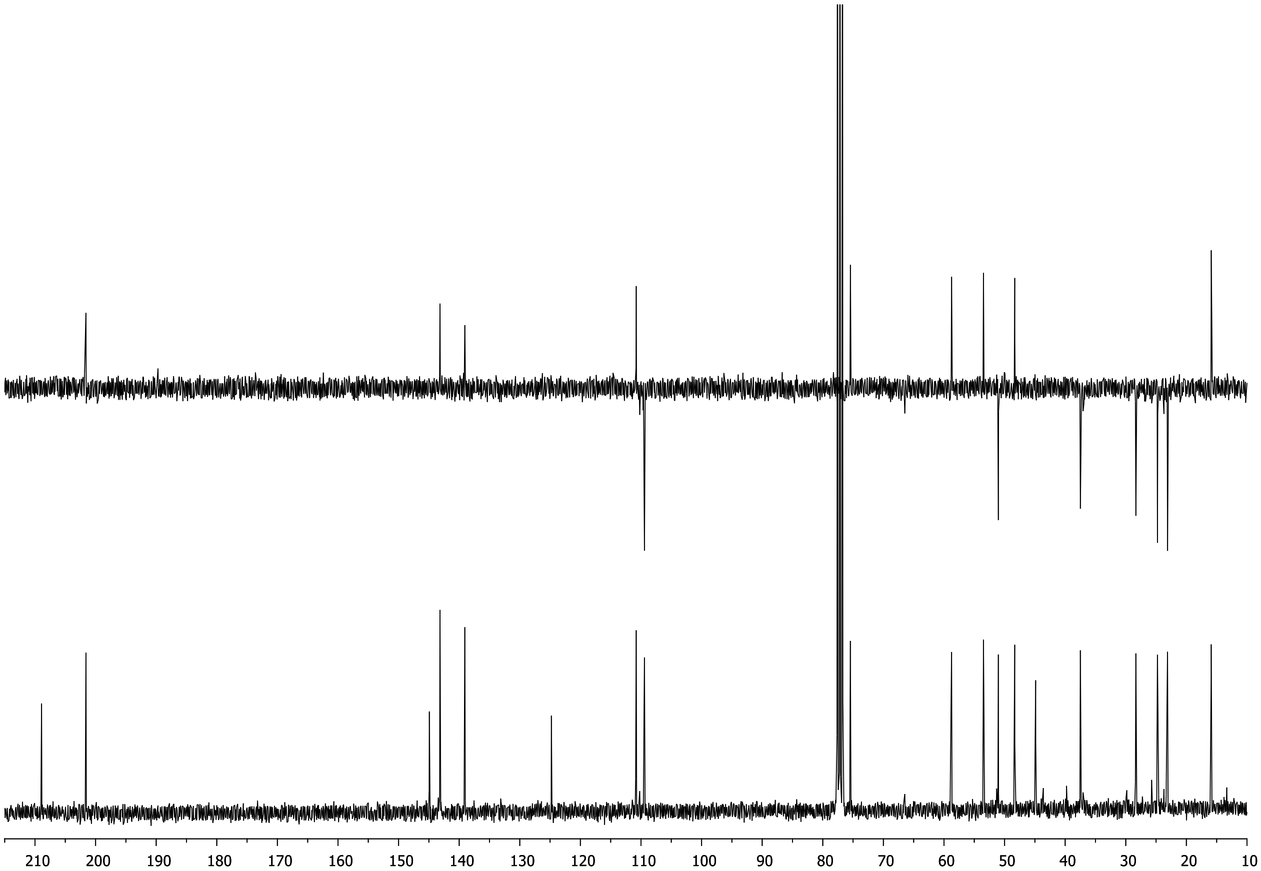

Figure S14. ^1H-^1H COSY spectrum of ($CDCl_3$) of compound **3**

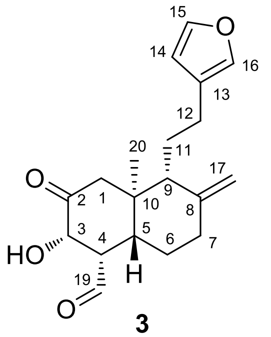

3

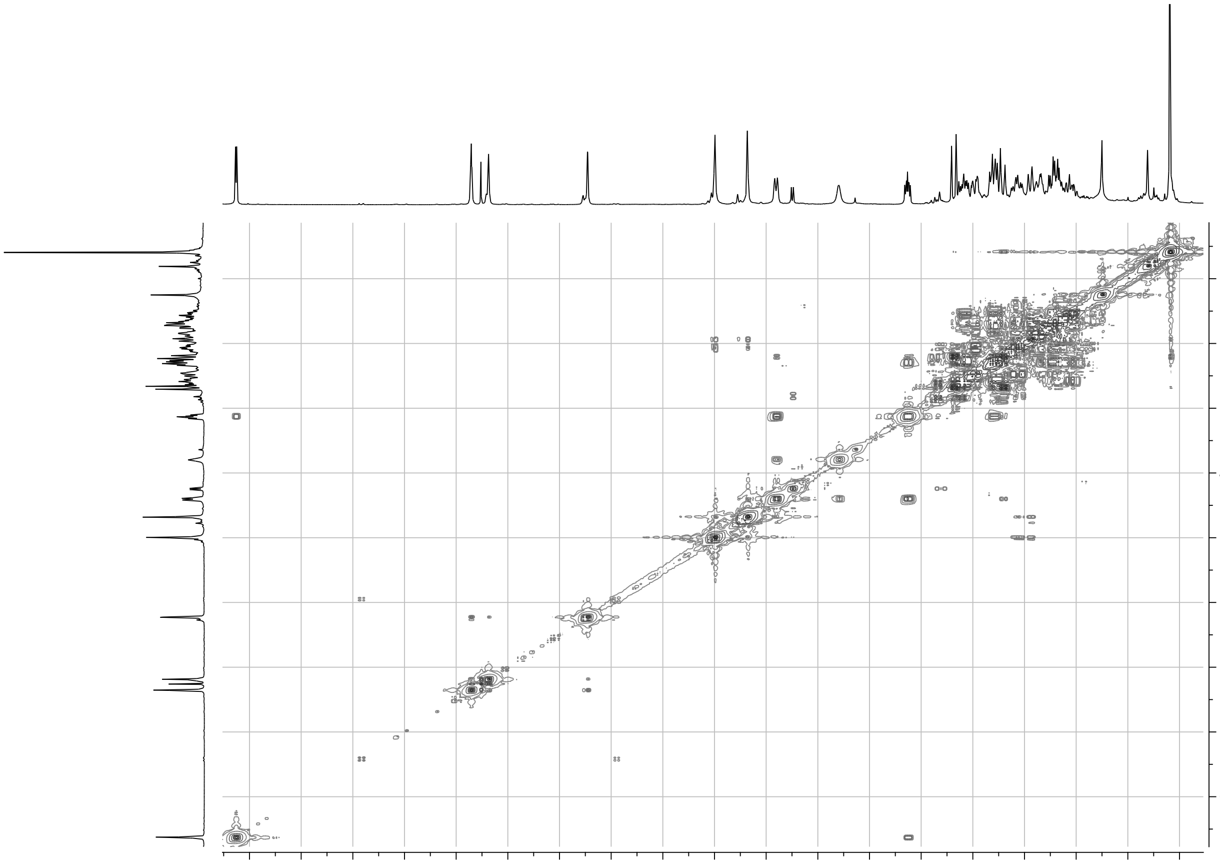

Figure S15. HMQC spectrum of ($CDCl_3$) of compound **3**

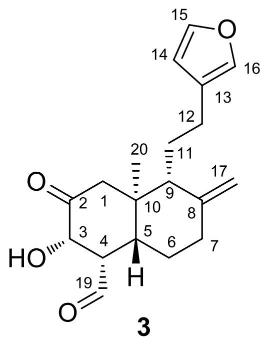

3

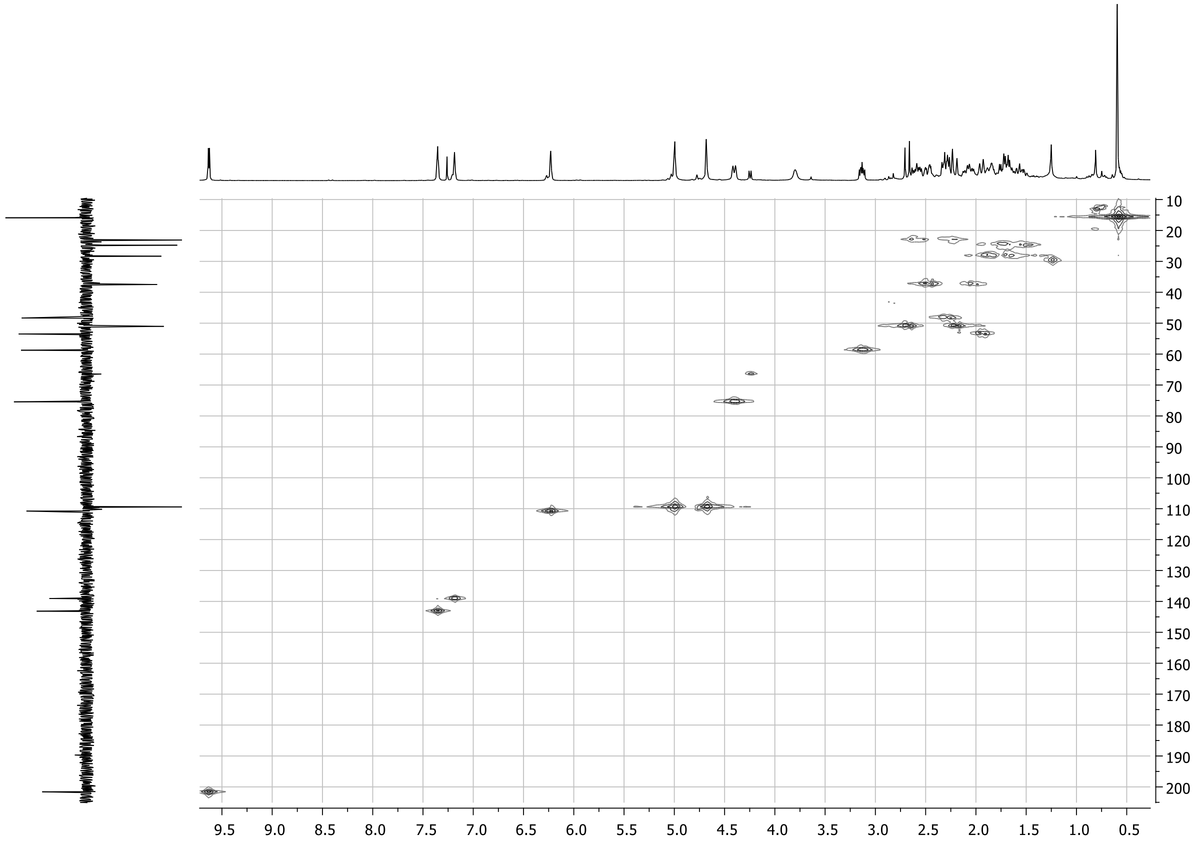

Figure S16. HMBC spectrum of ($CDCl_3$) of compound **3**

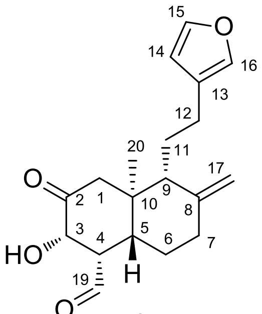

3

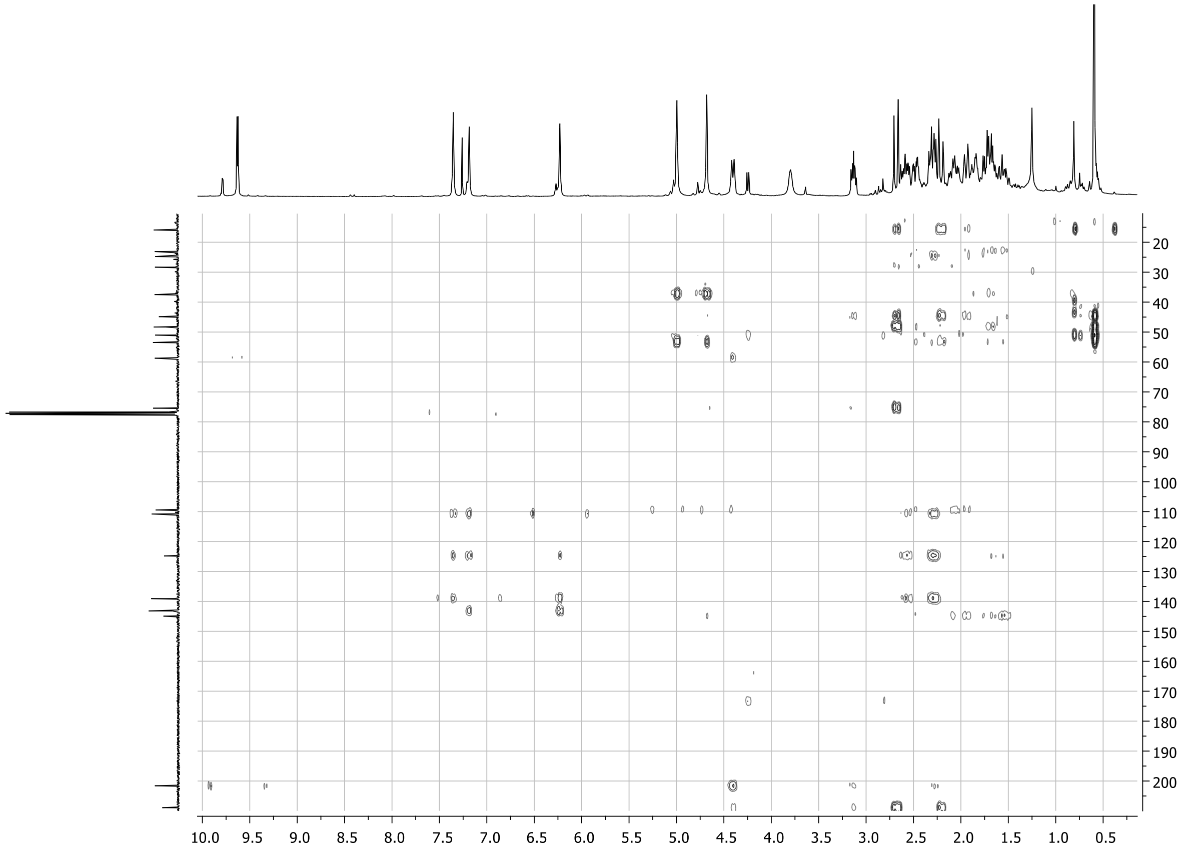

Figure S32. ^{1}H NMR spectrum (300 MHz, $CDCl_3$) of compound **4**

^{1}H NMR (300 MHz, $CDCl_3$, δ, ppm, J/Hz): 2.61 (d, J = 13.8, 3.4, H-1), 2.85 (d, J = 13.8, H-1’), 9.79 (d, J = 2.8, H-3), 2.81 (m, H-4), 1.84 (m, H-5), 1.78 (m, H-6), 2.03 (m, H-7), 2.40 (m, H-7’), 2.04 (m, H-9), 1.41 (m, H-11), 1.68 (m, H-11’), 2.27 (m, H-12), 2.60 (m, (H-12’), 6.27 (s broad, H-14), 7.36 (s broad, H-15), 7.21 (s, H-16), 4.78 (s, H-17), 5.03 (d, J = 0.90, H-17’4.25, (d, J = 5.9, H-19), 0.81 (s, H-20).

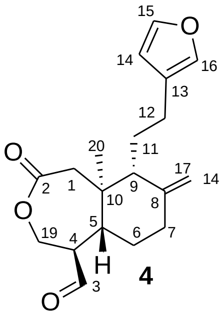

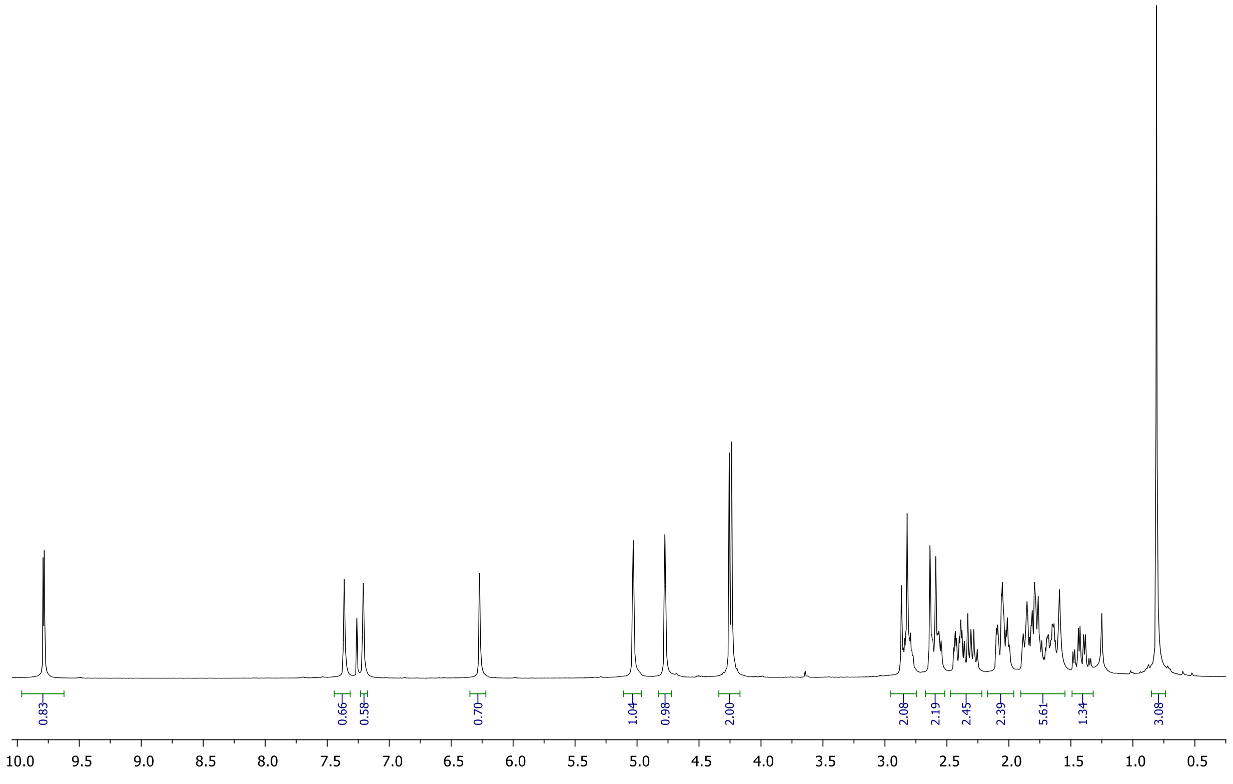

Figure S33. ^{13}C NMR spectrum (75 MHz, $CDCl_3$) of compound **4**

^{13}C NMR (75 MHz, $CDCl_3$, δ, ppm): 43.6 (C-1), 173.3 (C-2), 201.6 (C-3), 53.4 (C-4), 51.3 (C-5), 25.7 (C-6), 37.1 (C-7), 145.4 (C-8), 51.1 (C-9), 39.8 (C-10), 28.4 (C-11), 23.7 (C-12), 124.6 (C-13), 110.9 (C-14), 143.2 (C-15), 139.1 (C-16), 110.2 (C-17), 66.4 (C-19), 13.3 (C-20).

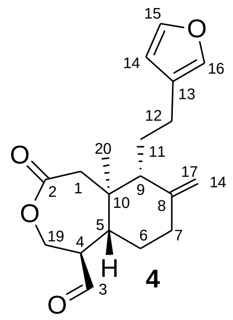

4

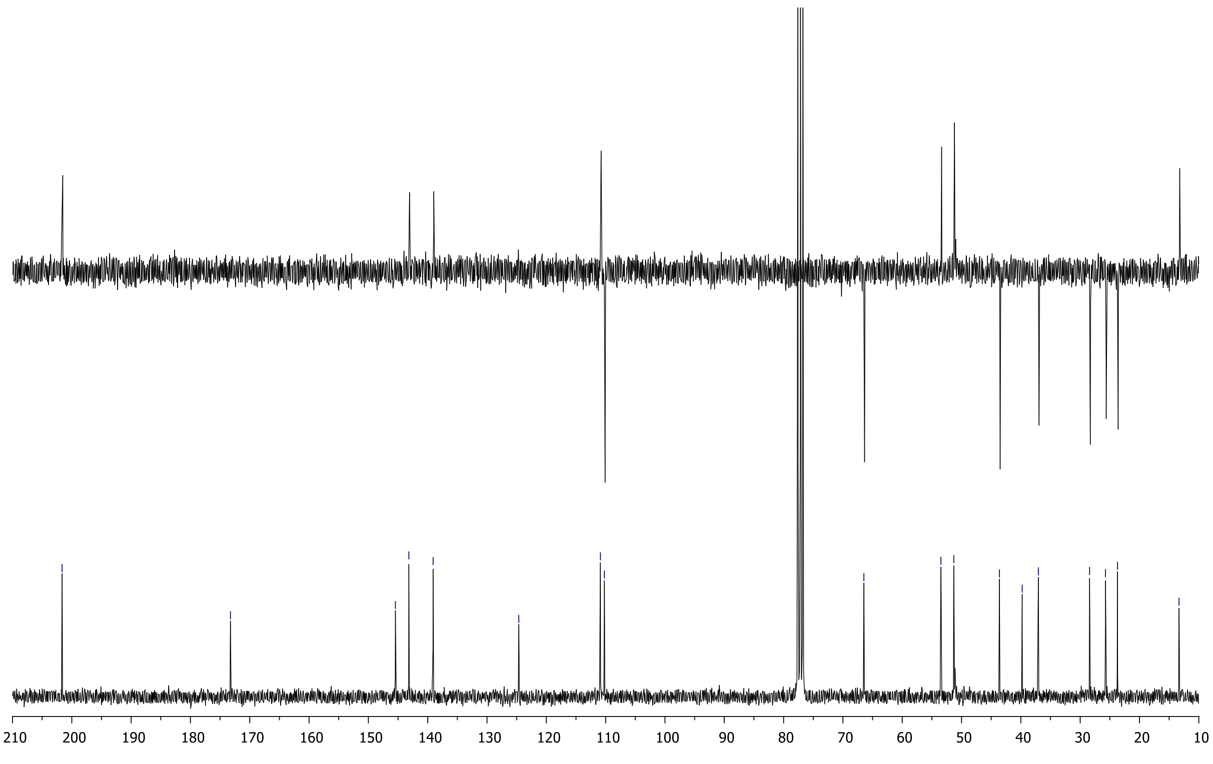

Figure S34. ^1H-^1H COSY spectrum of ($CDCl_3$) of compound **4**

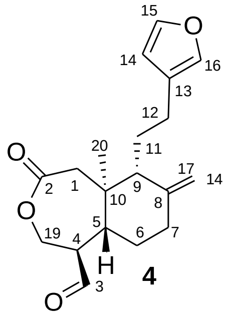

4

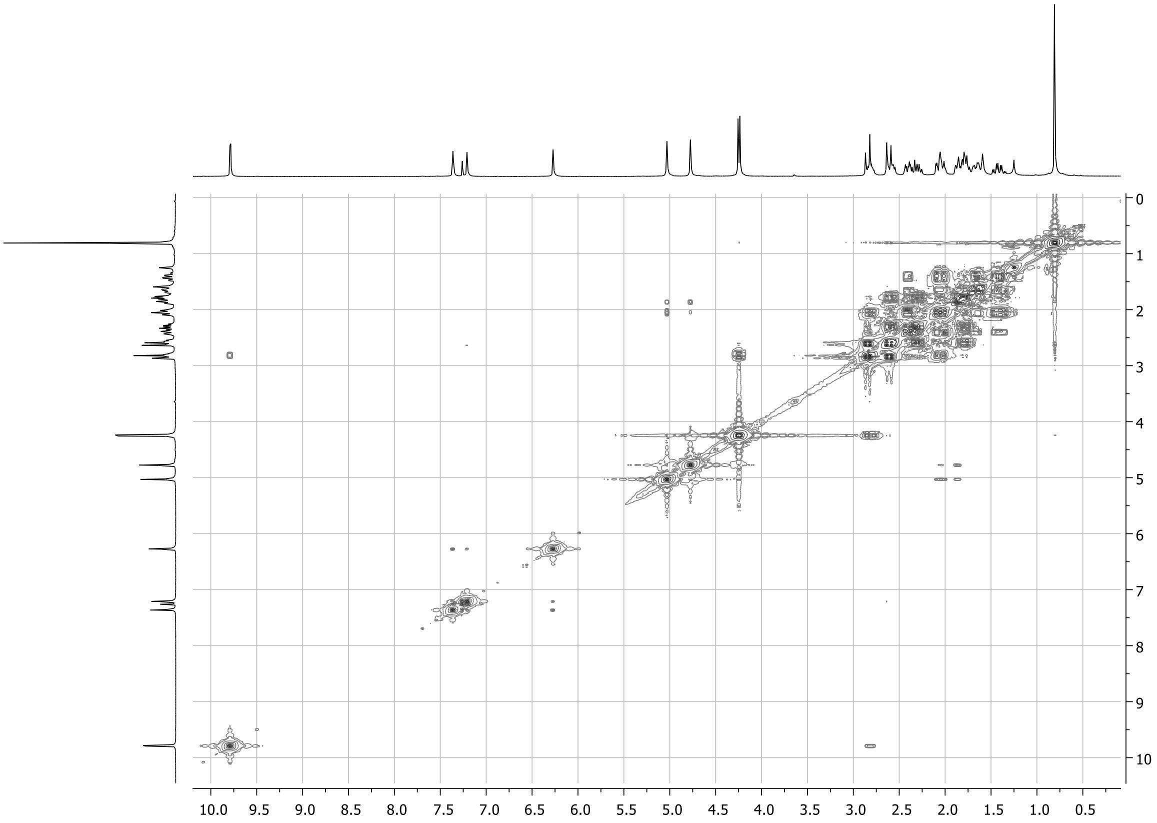

Figure S35. HMQC spectrum of ($CDCl_3$) of compound **4**

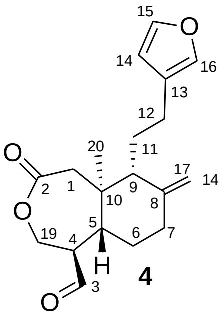

4

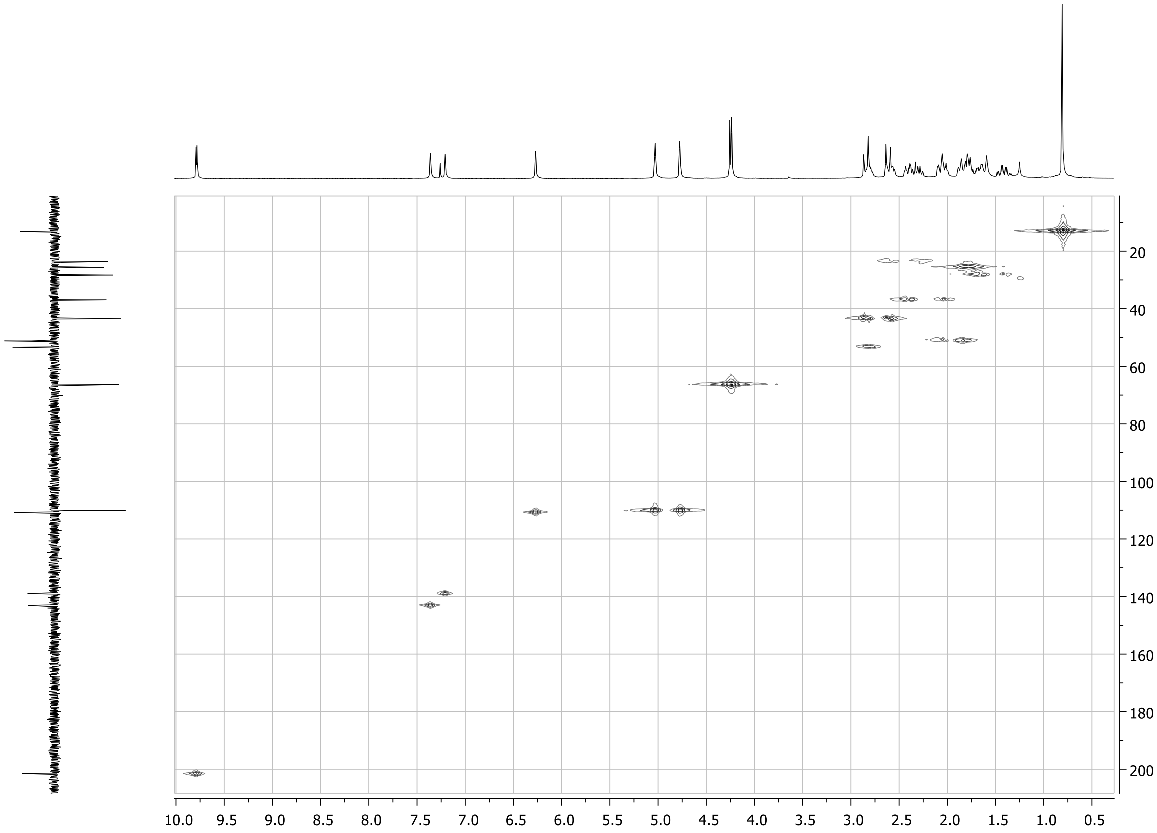

Figure S36. HMBC spectrum of ($CDCl_3$) of compound **4**

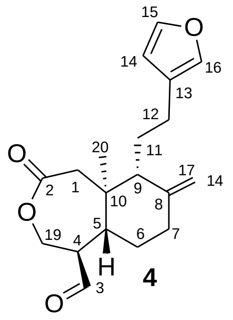

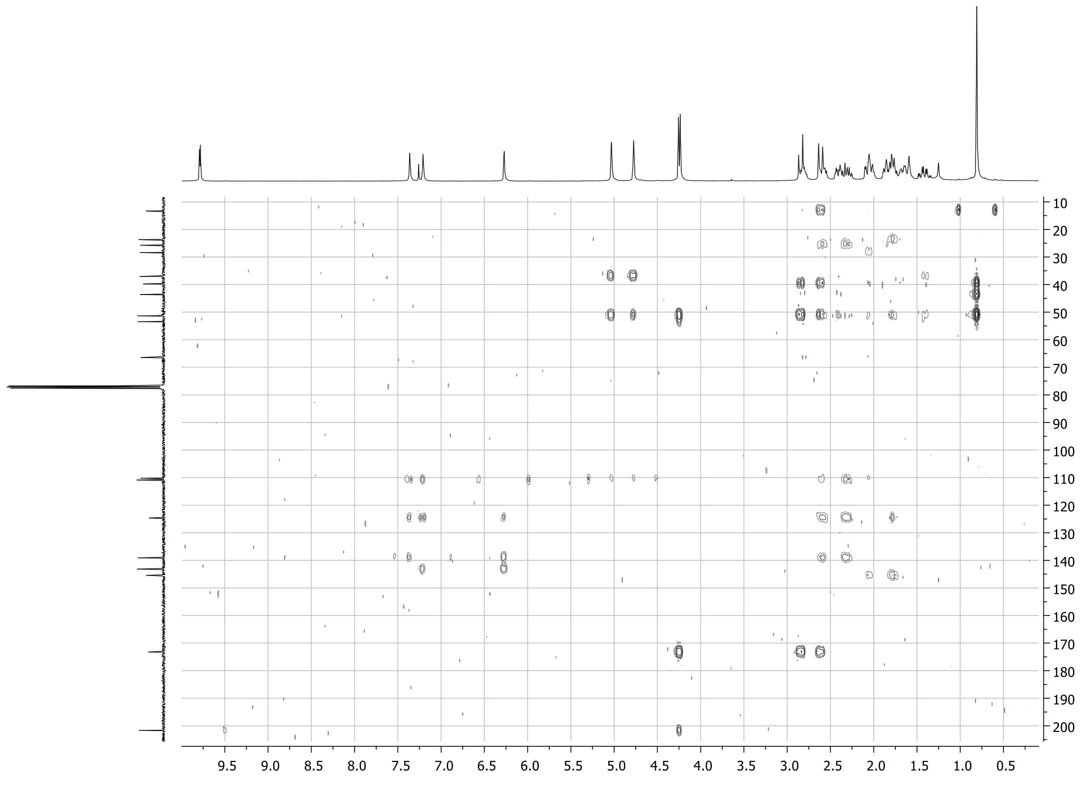

Supplement: Supplementary file 1 — Additional file 1. Reactions schemes. Additional figures S1–S21. [file 13659_2022_343_MOESM1_ESM.pdf]
